# Supplementary material for: Pyruvate Dehydrogenase Kinase 1 inhibition mediated oxidative phosphorylation enhancement in cartilage promotes osteoarthritis progression
Source: BMC Musculoskelet Disord. 2023 Jul 20;24:597. doi: 10.1186/s12891-023-06585-6 (PMC10357736; doi:10.1186/s12891-023-06585-6)
Supplement: Supplementary file 1 — Additional file 1. [file 12891_2023_6585_MOESM1_ESM.zip › BP.Gsea.1653623667859/GOBP_LIPOSACCHARIDE_METABOLIC_PROCESS.html]

Details for gene set GOBP\_LIPOSACCHARIDE\_METABOLIC\_PROCESS[GSEA]

|  || Dataset | 55457\_collapsed\_to\_symbols.cls.cls #OA\_versus\_normal.cls.cls #OA\_versus\_normal\_repos |
| Phenotype | cls.cls#OA\_versus\_normal\_repos |
| Upregulated in class | OA |
| GeneSet | GOBP\_LIPOSACCHARIDE\_METABOLIC\_PROCESS |
| Enrichment Score (ES) | 0.36849853 |
| Normalized Enrichment Score (NES) | 1.6524838 |
| Nominal p-value | 0.0 |
| FDR q-value | 0.21837665 |
| FWER p-Value | 1.0 |
Table: GSEA Results Summary

  

Fig 1: Enrichment plot: GOBP\_LIPOSACCHARIDE\_METABOLIC\_PROCESS      
 Profile of the Running ES Score & Positions of GeneSet Members on the Rank Ordered List

  

| SYMBOL | TITLE | RANK IN GENE LIST | RANK METRIC SCORE | RUNNING ES | CORE ENRICHMENT || 1 | GPAA1 | glycosylphosphatidylinositol anchor attachment 1 [Source:HGNC Symbol;Acc:HGNC:4446] | 44 | 1.006 | 0.0317 | Yes |
| 2 | B3GALNT1 | "beta-1,3-N-acetylgalactosaminyltransferase 1 (globoside blood group) [Source:HGNC Symbol;Acc:HGNC:918]" | 101 | 0.914 | 0.0593 | Yes |
| 3 | ST8SIA1 | "ST8 alpha-N-acetyl-neuraminide alpha-2,8-sialyltransferase 1 [Source:HGNC Symbol;Acc:HGNC:10869]" | 133 | 0.873 | 0.0874 | Yes |
| 4 | PIGC | phosphatidylinositol glycan anchor biosynthesis class C [Source:HGNC Symbol;Acc:HGNC:8960] | 202 | 0.816 | 0.1107 | Yes |
| 5 | GLB1 | galactosidase beta 1 [Source:HGNC Symbol;Acc:HGNC:4298] | 337 | 0.731 | 0.1257 | Yes |
| 6 | PIGP | phosphatidylinositol glycan anchor biosynthesis class P [Source:HGNC Symbol;Acc:HGNC:3046] | 367 | 0.718 | 0.1486 | Yes |
| 7 | PIGT | phosphatidylinositol glycan anchor biosynthesis class T [Source:HGNC Symbol;Acc:HGNC:14938] | 444 | 0.686 | 0.1666 | Yes |
| 8 | PIGO | phosphatidylinositol glycan anchor biosynthesis class O [Source:HGNC Symbol;Acc:HGNC:23215] | 462 | 0.675 | 0.1889 | Yes |
| 9 | GALC | galactosylceramidase [Source:HGNC Symbol;Acc:HGNC:4115] | 478 | 0.669 | 0.2111 | Yes |
| 10 | PIGF | phosphatidylinositol glycan anchor biosynthesis class F [Source:HGNC Symbol;Acc:HGNC:8962] | 798 | 0.570 | 0.2062 | Yes |
| 11 | B3GALT4 | "beta-1,3-galactosyltransferase 4 [Source:HGNC Symbol;Acc:HGNC:919]" | 962 | 0.526 | 0.2118 | Yes |
| 12 | PIGV | phosphatidylinositol glycan anchor biosynthesis class V [Source:HGNC Symbol;Acc:HGNC:26031] | 1064 | 0.503 | 0.2215 | Yes |
| 13 | SMPD1 | sphingomyelin phosphodiesterase 1 [Source:HGNC Symbol;Acc:HGNC:11120] | 1230 | 0.471 | 0.2251 | Yes |
| 14 | HEXB | hexosaminidase subunit beta [Source:HGNC Symbol;Acc:HGNC:4879] | 1434 | 0.438 | 0.2246 | Yes |
| 15 | B4GALT6 | "beta-1,4-galactosyltransferase 6 [Source:HGNC Symbol;Acc:HGNC:929]" | 1500 | 0.429 | 0.2345 | Yes |
| 16 | FUCA1 | alpha-L-fucosidase 1 [Source:HGNC Symbol;Acc:HGNC:4006] | 1506 | 0.428 | 0.2491 | Yes |
| 17 | TM9SF2 | transmembrane 9 superfamily member 2 [Source:HGNC Symbol;Acc:HGNC:11865] | 1582 | 0.416 | 0.2578 | Yes |
| 18 | PGAP2 | post-GPI attachment to proteins 2 [Source:HGNC Symbol;Acc:HGNC:17893] | 1669 | 0.403 | 0.2652 | Yes |
| 19 | NAGA | alpha-N-acetylgalactosaminidase [Source:HGNC Symbol;Acc:HGNC:7631] | 1674 | 0.402 | 0.2789 | Yes |
| 20 | MPPE1 | metallophosphoesterase 1 [Source:HGNC Symbol;Acc:HGNC:15988] | 1675 | 0.402 | 0.2930 | Yes |
| 21 | GBA3 | glucosylceramidase beta 3 (gene/pseudogene) [Source:HGNC Symbol;Acc:HGNC:19069] | 1767 | 0.388 | 0.2995 | Yes |
| 22 | ST8SIA4 | "ST8 alpha-N-acetyl-neuraminide alpha-2,8-sialyltransferase 4 [Source:HGNC Symbol;Acc:HGNC:10871]" | 1850 | 0.379 | 0.3063 | Yes |
| 23 | KIT | "KIT proto-oncogene, receptor tyrosine kinase [Source:HGNC Symbol;Acc:HGNC:6342]" | 2117 | 0.348 | 0.2977 | Yes |
| 24 | PRKCD | protein kinase C delta [Source:HGNC Symbol;Acc:HGNC:9399] | 2237 | 0.335 | 0.3001 | Yes |
| 25 | GM2A | GM2 ganglioside activator [Source:HGNC Symbol;Acc:HGNC:4367] | 2299 | 0.327 | 0.3068 | Yes |
| 26 | PGAP3 | post-GPI attachment to proteins phospholipase 3 [Source:HGNC Symbol;Acc:HGNC:23719] | 2322 | 0.324 | 0.3164 | Yes |
| 27 | PIGN | phosphatidylinositol glycan anchor biosynthesis class N [Source:HGNC Symbol;Acc:HGNC:8967] | 2484 | 0.304 | 0.3145 | Yes |
| 28 | PIGH | phosphatidylinositol glycan anchor biosynthesis class H [Source:HGNC Symbol;Acc:HGNC:8964] | 2573 | 0.294 | 0.3179 | Yes |
| 29 | DPM3 | "dolichyl-phosphate mannosyltransferase subunit 3, regulatory [Source:HGNC Symbol;Acc:HGNC:3007]" | 2618 | 0.289 | 0.3246 | Yes |
| 30 | CERK | ceramide kinase [Source:HGNC Symbol;Acc:HGNC:19256] | 2633 | 0.287 | 0.3335 | Yes |
| 31 | ST6GALNAC5 | "ST6 N-acetylgalactosaminide alpha-2,6-sialyltransferase 5 [Source:HGNC Symbol;Acc:HGNC:19342]" | 2641 | 0.286 | 0.3430 | Yes |
| 32 | PIGB | phosphatidylinositol glycan anchor biosynthesis class B [Source:HGNC Symbol;Acc:HGNC:8959] | 2663 | 0.284 | 0.3512 | Yes |
| 33 | PIGK | phosphatidylinositol glycan anchor biosynthesis class K [Source:HGNC Symbol;Acc:HGNC:8965] | 2801 | 0.270 | 0.3500 | Yes |
| 34 | B3GALT2 | "beta-1,3-galactosyltransferase 2 [Source:HGNC Symbol;Acc:HGNC:917]" | 2883 | 0.262 | 0.3528 | Yes |
| 35 | GAL3ST4 | galactose-3-O-sulfotransferase 4 [Source:HGNC Symbol;Acc:HGNC:24145] | 2979 | 0.252 | 0.3542 | Yes |
| 36 | HEXA | hexosaminidase subunit alpha [Source:HGNC Symbol;Acc:HGNC:4878] | 3025 | 0.247 | 0.3594 | Yes |
| 37 | SCCPDH | saccharopine dehydrogenase (putative) [Source:HGNC Symbol;Acc:HGNC:24275] | 3197 | 0.231 | 0.3541 | Yes |
| 38 | PNLIPRP2 | pancreatic lipase related protein 2 (gene/pseudogene) [Source:HGNC Symbol;Acc:HGNC:9157] | 3268 | 0.224 | 0.3565 | Yes |
| 39 | GBA | glucosylceramidase beta [Source:HGNC Symbol;Acc:HGNC:4177] | 3327 | 0.219 | 0.3596 | Yes |
| 40 | ST3GAL6 | "ST3 beta-galactoside alpha-2,3-sialyltransferase 6 [Source:HGNC Symbol;Acc:HGNC:18080]" | 3339 | 0.218 | 0.3664 | Yes |
| 41 | B4GALT4 | "beta-1,4-galactosyltransferase 4 [Source:HGNC Symbol;Acc:HGNC:927]" | 3407 | 0.211 | 0.3685 | Yes |
| 42 | ST3GAL5 | "ST3 beta-galactoside alpha-2,3-sialyltransferase 5 [Source:HGNC Symbol;Acc:HGNC:10872]" | 3667 | 0.188 | 0.3549 | No |
| 43 | CLN6 | CLN6 transmembrane ER protein [Source:HGNC Symbol;Acc:HGNC:2077] | 3678 | 0.188 | 0.3607 | No |
| 44 | AOAH | acyloxyacyl hydrolase [Source:HGNC Symbol;Acc:HGNC:548] | 3978 | 0.158 | 0.3428 | No |
| 45 | ST8SIA2 | "ST8 alpha-N-acetyl-neuraminide alpha-2,8-sialyltransferase 2 [Source:HGNC Symbol;Acc:HGNC:10870]" | 4153 | 0.142 | 0.3342 | No |
| 46 | GLA | galactosidase alpha [Source:HGNC Symbol;Acc:HGNC:4296] | 4172 | 0.140 | 0.3377 | No |
| 47 | NEU1 | neuraminidase 1 [Source:HGNC Symbol;Acc:HGNC:7758] | 4486 | 0.114 | 0.3173 | No |
| 48 | CPTP | ceramide-1-phosphate transfer protein [Source:HGNC Symbol;Acc:HGNC:28116] | 4654 | 0.100 | 0.3077 | No |
| 49 | PIGQ | phosphatidylinositol glycan anchor biosynthesis class Q [Source:HGNC Symbol;Acc:HGNC:14135] | 4825 | 0.085 | 0.2975 | No |
| 50 | DPM2 | "dolichyl-phosphate mannosyltransferase subunit 2, regulatory [Source:HGNC Symbol;Acc:HGNC:3006]" | 4862 | 0.083 | 0.2975 | No |
| 51 | GPLD1 | glycosylphosphatidylinositol specific phospholipase D1 [Source:HGNC Symbol;Acc:HGNC:4459] | 5103 | 0.064 | 0.2810 | No |
| 52 | ABCA2 | ATP binding cassette subfamily A member 2 [Source:HGNC Symbol;Acc:HGNC:32] | 5349 | 0.044 | 0.2635 | No |
| 53 | FA2H | fatty acid 2-hydroxylase [Source:HGNC Symbol;Acc:HGNC:21197] | 5466 | 0.035 | 0.2557 | No |
| 54 | NEU2 | neuraminidase 2 [Source:HGNC Symbol;Acc:HGNC:7759] | 5775 | 0.010 | 0.2320 | No |
| 55 | B3GALT1 | "beta-1,3-galactosyltransferase 1 [Source:HGNC Symbol;Acc:HGNC:916]" | 5864 | 0.004 | 0.2252 | No |
| 56 | UGT8 | UDP glycosyltransferase 8 [Source:HGNC Symbol;Acc:HGNC:12555] | 5908 | 0.002 | 0.2219 | No |
| 57 | PIGG | phosphatidylinositol glycan anchor biosynthesis class G [Source:HGNC Symbol;Acc:HGNC:25985] | 6452 | -0.039 | 0.1809 | No |
| 58 | ST8SIA5 | "ST8 alpha-N-acetyl-neuraminide alpha-2,8-sialyltransferase 5 [Source:HGNC Symbol;Acc:HGNC:17827]" | 6565 | -0.047 | 0.1738 | No |
| 59 | CWH43 | cell wall biogenesis 43 C-terminal homolog [Source:HGNC Symbol;Acc:HGNC:26133] | 6648 | -0.053 | 0.1693 | No |
| 60 | GLTP | glycolipid transfer protein [Source:HGNC Symbol;Acc:HGNC:24867] | 7113 | -0.089 | 0.1362 | No |
| 61 | LARGE1 | LARGE xylosyl- and glucuronyltransferase 1 [Source:HGNC Symbol;Acc:HGNC:6511] | 7164 | -0.093 | 0.1355 | No |
| 62 | DPM1 | "dolichyl-phosphate mannosyltransferase subunit 1, catalytic [Source:HGNC Symbol;Acc:HGNC:3005]" | 7723 | -0.135 | 0.0967 | No |
| 63 | B4GALNT1 | "beta-1,4-N-acetyl-galactosaminyltransferase 1 [Source:HGNC Symbol;Acc:HGNC:4117]" | 7799 | -0.141 | 0.0958 | No |
| 64 | GAL3ST1 | galactose-3-O-sulfotransferase 1 [Source:HGNC Symbol;Acc:HGNC:24240] | 8378 | -0.184 | 0.0571 | No |
| 65 | PIGZ | phosphatidylinositol glycan anchor biosynthesis class Z [Source:HGNC Symbol;Acc:HGNC:30596] | 9047 | -0.235 | 0.0132 | No |
| 66 | B4GALT5 | "beta-1,4-galactosyltransferase 5 [Source:HGNC Symbol;Acc:HGNC:928]" | 9051 | -0.236 | 0.0213 | No |
| 67 | BAX | "BCL2 associated X, apoptosis regulator [Source:HGNC Symbol;Acc:HGNC:959]" | 9323 | -0.257 | 0.0091 | No |
| 68 | PGAP4 | post-GPI attachment to proteins GalNAc transferase 4 [Source:HGNC Symbol;Acc:HGNC:28180] | 9347 | -0.259 | 0.0164 | No |
| 69 | NEU3 | neuraminidase 3 [Source:HGNC Symbol;Acc:HGNC:7760] | 9348 | -0.259 | 0.0254 | No |
| 70 | PGAP1 | post-GPI attachment to proteins inositol deacylase 1 [Source:HGNC Symbol;Acc:HGNC:25712] | 9358 | -0.260 | 0.0338 | No |
| 71 | FUT9 | fucosyltransferase 9 [Source:HGNC Symbol;Acc:HGNC:4020] | 9433 | -0.268 | 0.0374 | No |
| 72 | ITGB8 | integrin subunit beta 8 [Source:HGNC Symbol;Acc:HGNC:6163] | 9442 | -0.268 | 0.0461 | No |
| 73 | PIGA | phosphatidylinositol glycan anchor biosynthesis class A [Source:HGNC Symbol;Acc:HGNC:8957] | 9571 | -0.280 | 0.0459 | No |
| 74 | A4GALT | "alpha 1,4-galactosyltransferase (P blood group) [Source:HGNC Symbol;Acc:HGNC:18149]" | 9766 | -0.297 | 0.0412 | No |
| 75 | ST8SIA3 | "ST8 alpha-N-acetyl-neuraminide alpha-2,8-sialyltransferase 3 [Source:HGNC Symbol;Acc:HGNC:14269]" | 10046 | -0.326 | 0.0308 | No |
| 76 | ST3GAL4 | "ST3 beta-galactoside alpha-2,3-sialyltransferase 4 [Source:HGNC Symbol;Acc:HGNC:10864]" | 10688 | -0.391 | -0.0055 | No |
| 77 | ST6GALNAC4 | "ST6 N-acetylgalactosaminide alpha-2,6-sialyltransferase 4 [Source:HGNC Symbol;Acc:HGNC:17846]" | 11164 | -0.452 | -0.0268 | No |
| 78 | FUT2 | fucosyltransferase 2 [Source:HGNC Symbol;Acc:HGNC:4013] | 11459 | -0.497 | -0.0323 | No |
| 79 | PRKAA1 | protein kinase AMP-activated catalytic subunit alpha 1 [Source:HGNC Symbol;Acc:HGNC:9376] | 11499 | -0.501 | -0.0179 | No |
| 80 | ST3GAL2 | "ST3 beta-galactoside alpha-2,3-sialyltransferase 2 [Source:HGNC Symbol;Acc:HGNC:10863]" | 11721 | -0.536 | -0.0163 | No |
| 81 | B4GALT3 | "beta-1,4-galactosyltransferase 3 [Source:HGNC Symbol;Acc:HGNC:926]" | 11748 | -0.540 | 0.0005 | No |
| 82 | PIGL | phosphatidylinositol glycan anchor biosynthesis class L [Source:HGNC Symbol;Acc:HGNC:8966] | 11817 | -0.553 | 0.0146 | No |
| 83 | FUT6 | fucosyltransferase 6 [Source:HGNC Symbol;Acc:HGNC:4017] | 12067 | -0.612 | 0.0165 | No |
| 84 | ST3GAL1 | "ST3 beta-galactoside alpha-2,3-sialyltransferase 1 [Source:HGNC Symbol;Acc:HGNC:10862]" | 12092 | -0.619 | 0.0363 | No |
| 85 | UGCG | UDP-glucose ceramide glucosyltransferase [Source:HGNC Symbol;Acc:HGNC:12524] | 12532 | -0.763 | 0.0287 | No |
Table: GSEA details [plain text format]

  

Fig 2: GOBP\_LIPOSACCHARIDE\_METABOLIC\_PROCESS      
 Blue-Pink O' Gram in the Space of the Analyzed GeneSet

  

Fig 3: GOBP\_LIPOSACCHARIDE\_METABOLIC\_PROCESS: Random ES distribution      
 Gene set null distribution of ES for **GOBP\_LIPOSACCHARIDE\_METABOLIC\_PROCESS**
